# Implications of Cross-Reactivity and Cross-Protection for Pneumococcal Vaccine Development

**DOI:** 10.3390/vaccines12090974

**Published:** 2024-08-28

**Authors:** Kristen Feemster, William P. Hausdorff, Natalie Banniettis, Heather Platt, Priscilla Velentgas, Alejandra Esteves-Jaramillo, Robert L. Burton, Moon H. Nahm, Ulrike K. Buchwald

**Affiliations:** 1 Merck & Co., Inc., Rahway, NJ 07065, USA; natalie.banniettis@merck.com (N.B.); heather.platt@merck.com (H.P.); priscilla.velentgas@merck.com (P.V.); alex.esteves@merck.com (A.E.-J.); ulrike.buchwald@merck.com (U.K.B.); 2 Center for Vaccine Innovation and Access, PATH, 455 Massachusetts Ave NW, Washington, DC 20001, USA; whausdorff@path.org; 3 Faculty of Medicine, Université Libre de Bruxelles, 1050 Brussels, Belgium; 4 SunFire Biotechnologies LLC, Birmingham, AL 35203, USA; rob@sunfirebio.com; 5 Heersink School of Medicine, University of Alabama at Birmingham, Birmingham, AL 35233, USA; mnahm@uabmc.edu

**Keywords:** cross-reactivity, cross-protection, pneumococcal vaccines, PCVs, *Streptococcus pneumoniae*

## Abstract

Pneumococcal vaccines are a cornerstone for the prevention of pneumococcal diseases, reducing morbidity and mortality in children and adults worldwide. Pneumococcal vaccine composition is based on the polysaccharide capsule of *Streptococcus pneumoniae*, which is one of the most important identified contributors to the pathogen’s virulence. Similarities in the structural composition of polysaccharides included in licensed pneumococcal vaccines may result in cross-reactivity of immune response against closely related serotypes, including serotypes not included in the vaccine. Therefore, it is important to understand whether cross-reactive antibodies offer clinical protection against pneumococcal disease. This review explores available evidence of cross-reactivity and cross-protection associated with pneumococcal vaccines, the challenges associated with the assessment of cross-reactivity and cross-protection, and implications for vaccine design and development.

## 1. Introduction

*Streptococcus pneumoniae* is a Gram-positive, facultatively anaerobic bacterium that commonly colonizes the respiratory tract of people, especially children. It is spread through respiratory droplets and can cause a wide range of invasive (infection of normally sterile spaces, including bacteremic pneumonia, bacteremia, meningitis, septic arthritis, and osteomyelitis) and non-invasive infections (including non-bacteremic pneumonia, otitis media, and sinusitis) [1].

Prevention of pneumococcal carriage and disease is based on the use of pneumococcal vaccines, while prophylactic antibiotics are used rarely in specific situations [2]. All currently available pneumococcal vaccines include polysaccharides extracted from the capsules of vaccine serotypes, which are a characteristic of most disease-causing strains of *S. pneumoniae* [3,4], and elicit protective antibodies against the specific serotypes included in the vaccines [1,5]. The 23-valent pneumococcal polysaccharide vaccine (PPSV23; PNEUMOVAX^®^ 23, Merck Sharp & Dohme LLC, a subsidiary of Merck & Co., Inc., Rahway, NJ, USA) has effectively lowered the incidence and burden of invasive pneumococcal disease (IPD) in vaccinated older adults and individuals with at-risk or high-risk conditions [6]; however, polysaccharide vaccines have limited immunogenicity in young infants [7]. Pneumococcal conjugate vaccines (PCVs), which are comprised of capsular polysaccharides conjugated to carrier proteins to provide immunological memory in young infants, were subsequently developed and have been widely used to reduce the burden of pediatric disease [8]. The first PCV to be introduced in the United States was a seven-valent PCV, comprised of seven polysaccharides conjugated to diphtheria toxoid cross-reactive material 197 (CRM_197_) (PCV7-CRM; Prevnar™; Wyeth LLC, marketed by Pfizer, New York, NY, USA) [1,9]. This was followed by a 13-valent PCV (PCV13-CRM; Prevnar 13™; Wyeth LLC, marketed by Pfizer, New York, NY, USA) in 2009/2010, a 15-valent PCV (PCV15-CRM; VAXNEUVANCE™, Merck Sharp & Dohme LLC, a subsidiary of Merck & Co., Inc., Rahway, NJ, USA) in 2021, a 20-valent PCV (PCV20-CRM; Prevnar 20^®^; Wyeth LLC, marketed by Pfizer, New York, NY, USA) in 2021, and a 21-valent PCV (PCV21-CRM; CAPVAXIVE^TM^, Merck Sharp & Dohme LLC, a subsidiary of Merck & Co., Inc., Rahway, NJ, USA) in 2024 [10,11,12,13,14]. In addition, there are PCVs that have been licensed outside of the United States, including a 10-valent PCV with mixed carrier proteins (PCV10-mixed; Synflorix^TM^, GlaxoSmithKline Biologicals SA, Rixensart, Belgium) and a 10-valent pneumococcal polysaccharide conjugate vaccine, PCV10-CRM (Pneumosil^®^, Serum Institute of India, Pune, India), both of which have received World Health Organization (WHO) prequalification (in addition to PCV13) [15,16,17,18].

Pneumococcal vaccines have demonstrated effectiveness in preventing IPD and, to a lesser extent, non-invasive disease, including non-bacteremic pneumonia and acute otitis media (AOM) [1,19]. PCVs are also effective in reducing the frequency of acquisition of nasopharyngeal colonization and, consequently, transmission between humans. This indirect impact has contributed substantially to the overall public health impact of PCVs [1,19]. However, pneumococcal disease still causes considerable morbidity and mortality, particularly among infants < 2 years of age and older adults [20,21].

Owing to the challenges associated with monitoring non-invasive disease, pneumococcal disease surveillance generally focuses on IPD (i.e., the identification of pneumococci in sterile body fluids), which is a reportable disease in many countries. In Europe and the United States, recent reported incidence rates for IPD in infants < 1 year of age were 7.4 and 6.1 cases per 100,000 people, respectively. While these rates are considerably lower than those observed in the pre-PCV era, they still reflect a substantial burden of disease, despite widespread use of PCVs [20,21,22]. Similarly, a high incidence of IPD was seen among adults, especially those >65 years of age in Europe (6.61 cases per 100,000 people) and the United States (11.0, 12.4, and 16.8 cases per 100,000 people in the 65–74, 75–84, and ≥85 years age groups, respectively) [20,21]. High incidences of pneumococcal disease have also been reported in many other countries worldwide [23].

The distinct chemical composition and immunological properties of the capsular polysaccharide define pneumococcal serotypes [3]; antigenically related serotypes are categorized into serogroups [4]. These capsular polysaccharides consist primarily of oligosaccharide repeating units, the chemical structures of which are largely known [3,4]. The polysaccharide capsule is one of the most important virulence factors for pneumococci and aids the bacteria in immune escape [3,4]. Serotypes that colonize the nasopharynx also vary in prevalence, density, and duration, as well as in their capacity to cause mucosal disease or invasive disease (invasiveness) [24,25]. The likelihood that a colonizing serotype will cause invasive disease, or serotype invasiveness, is quantified as a case-to-carrier ratio [24].

More than 100 immunologically distinct pneumococcal serotypes are known; however, only a select number of serotypes are responsible for the majority of invasive disease [1,26]. Differences in the distribution of serogroups and serotypes are observed across geographic regions, age groups, and time periods [1,27,28,29], including regions with comparable socioeconomic conditions, such as North America and Western Europe [27]. Regional variability is thought to be due to several factors, including antibiotic use patterns as well as the differential uptake and impact of PCV use [19,30]. Serotyping of disease-causing pneumococcal isolates is an essential component of surveillance and epidemiological studies, providing vital information for vaccine development and determining the impact of serotype-specific pneumococcal vaccines [31]. However, it is important to note that the identification of serotypes is an evolving field and is based on currently available serotyping techniques [31,32].

As the capsules of some pneumococcal serotypes are similar in composition and structure, polysaccharide antigens of some vaccine serotypes can elicit cross-reactive antibodies to closely related serotypes, including non-vaccine serotypes [33]. Cross-reactivity describes the phenomenon in which immune recognition molecules, such as antibodies, directed against one specific antigen (e.g., following immunization with a vaccine antigen), bind to a different antigen (e.g., a non-vaccine antigen) [34,35]. This concept has been well documented between related pneumococcal capsules and proteins [7,36], as well as influenza glycoproteins [37,38], human papillomavirus [39], and haptens [40,41]. In some instances, vaccine-induced antibodies may also cross-react with human proteins [7], food [42], and commensal microorganisms [43]. When assessing the potential impact of vaccines, it is important to consider whether cross-reactive immune responses, such as cross-reactive antibodies, are also protecting against disease. Cross-protection is considered to be present when an immune response elicited with an organism or vaccine results in measurable protection against disease or infection by non-vaccine strains (or types), as assessed by incidence rates or disease severity [33].

In this review, we discuss published evidence of cross-reactivity and cross-protection in pneumococcal vaccines and how this evidence has informed vaccine design and development.

## 2. Methods

The objective of this narrative review was to summarize clinical trial data and real-world evidence that has explored cross-reactivity and cross-protection across all historical or currently licensed pneumococcal vaccines. A literature search was performed in PubMed for English-language articles published between January 1998 and June 2024, exploring cross-reactivity and/or cross protection in humans.

The primary outcomes for this review were evidence of cross-reactivity and/or cross-protection associated with the use of pneumococcal vaccines against related serotypes within a serogroup. Epidemiologically important pneumococcal serotypes that showed potential for cross-reactivity due to similarities in their polysaccharide structures were highlighted. Serotypes of particular interest were serotypes 6A/6B/6C/6D, 9A/9N/9L/9V, 15A/15B/15C/15F, 19A/19F, 20A/20B, and 23A/23B/23F. When there were a lack of adequate human data for the pre-identified serotypes of interest, animal data were included, where available.

## 3. The Basis of Cross-Reactivity and Cross-Protection

The primary aim of vaccination is to protect populations against diseases by stimulating robust humoral and cellular protective immune responses against antigens included in the vaccine [33]. Antigens of microorganisms typically differ in their immunogenic and structural characteristics, but some antigens may exhibit shared features, including common epitopes (defined as the area of an antigen that is recognized by a specific antibody or T-cell receptor) [34]. These shared epitopes are often found in closely related members of a pathogen species, but as noted earlier, they can also exist among more distantly related variants of the same species or even between different species. Vaccine-induced immune responses, including antibodies, can exhibit cross-reactivity among pathogens that carry the same antigenic determinants and epitopes [33]. These cross-reactive responses serve as the foundation for cross-protection associated with vaccination, where clinical protection extends beyond the primary pathogen the vaccine is directed against.

The evaluation of vaccine-induced immunity often focuses on antibody responses, for which well-characterized, scalable assays are generally more technically feasible than for cellular/functional responses. Cross-reactivity is an intrinsic characteristic of humoral immune responses, as each antibody contains several sites (paratopes) that physically bind to epitopes. Immune responses to antigens are typically polyclonal and contain high-specificity and lower-specificity interactions; epitope–paratope interactions strengthen and become more specific during affinity maturation [33,34].

**Figure 1:**
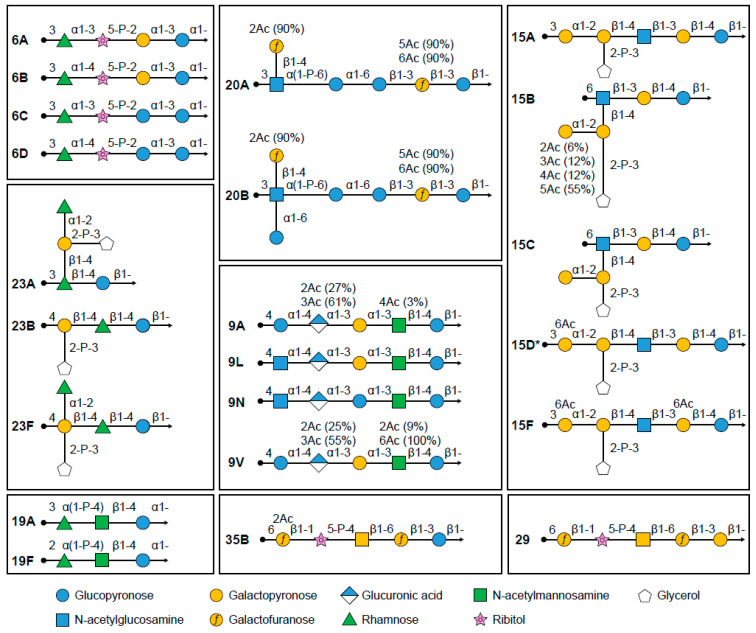
Polysaccharide structures of certain pneumococcal serotypes. * Predicted structure of serotype 15D.

Cross-reactive antibodies following pneumococcal vaccination are most commonly found between the closely related serotypes that are categorized in one serogroup. The difference in composition and structure of some serotypes within a serogroup can be very small; for example, the capsular polysaccharides found in serotypes 6A, 6B, and 6C exhibit linear polymer structures and are extremely similar (Figure 1). Polymer structures for 6A and 6B are identical, except for a difference in the linkage between rhamnose and ribitol, and 6C is also similar to 6A, except for replacing a glucose molecule with a galactose (Figure 1) [44].

Based on the intrinsic features of antibody–antigen interactions, the robustness of cross-reactive antibodies within a serogroup can differ based on the serotype construct included in the vaccine [5,45,46,47]. For example, PCV7-CRM, which contains serotype 6B [9], exhibited protection against serotype 6A, but much less protection against 6C [5]. By contrast, PCV13-CRM, which contains serotypes 6A and 6B [10], induced cross-reactive antibodies with greater functional activity (measured by opsonophagocytic activity (OPA) response) against serotype 6C compared with those induced by PCV7-CRM, attributed to the inclusion of serotype 6A in addition to serotype 6B [5,45].

Interestingly, cross-reactivity can also occur between serotypes in different serogroups: for example, Dob1, a human immunoglobulin IgG2 hybridoma against the polysaccharide of serotype 6B, secretes an antibody that cross-reacts with four serotypes (6A, 6B, 6C, and 19A) [48]. In principle, passive immunization using the Dob1 epitope has the potential to protect against these three serotypes in serogroup 6 as well as 19A [48]. Recent experiments displayed cross-reactivity against serotype 29 in mice that were vaccinated with monovalent serotype 35B PCV conjugated to CRM_197_, implying the possibility of immune protection extending from serogroup 35 to serogroup 29 [49]. However, there is limited clinical evidence on the impact of cross-reactive antibodies on disease caused by serotypes in different serogroups [50].

## 4. Challenges of Assessing Cross-Reactivity and Cross-Protection of Pneumococcal Vaccines

### 4.1. Serotype Classification

The methodology of serotype classification is evolving in a way that may affect several surveillance activities, including the detection and reporting of pneumococcal disease attributable to specific serotypes (e.g., serogroup 15 isolates are often reported as 15B/C, as they interconvert and can be difficult to distinguish) [51,52]. Therefore, there is a need for an efficient and specific method to detect and distinguish between pneumococcal serotypes. Molecular techniques for identifying capsule types, such as multiplex polymerase chain reaction (PCR), have been widely adopted in reference laboratories as a replacement for the traditional Quellung method. These methods are favored by many laboratories owing to their faster results and lower costs. Although various PCR methods focus on frequently encountered serotypes linked to vaccines, they unfortunately do not yet encompass all serotypes. Moreover, some closely related serotypes with similar genetic capsule structures cannot be differentiated using the PCR methods [53]. Consequently, many epidemiologic surveys often use whole-genome sequencing of an isolate to deduce its serotype, with different whole-genome sequencing kits readily available [54,55]. Whole-genome sequencing assays present several benefits compared with serological methods, including simplified interpretation and the ability to handle multiple targets simultaneously; moreover, in certain instances, culture may not be required. Nonetheless, whole-genome sequencing also has limitations, particularly when applied in surveillance contexts. Genetic similarities among certain serotypes hinder complete resolution of all serogroups, and as noted for PCR, most currently available assays only cover prevalent serotypes, leading to an incomplete detection of all existing serotypes [53].

### 4.2. Assessment of Immune Response

Assessment of vaccine-generated protective immunity typically includes measurements of binding or functional antibody levels; binding antibodies are commonly determined with techniques such as enzyme-linked immunosorbent assay (ELISA) [56,57] and functional immune responses through neutralization, opsonizing, or bactericidal assays [58,59]. For pneumococcal vaccines, functionality of antibodies is based on the measurement of the opsonic ability of vaccine-induced antibodies in specific OPA assays, as pneumococcal antibodies protect the host through opsonization [60,61]. ‘Cross-opsonic’ activity occurs when vaccine serotypes closely related to other pneumococcal serotypes confer not just binding but also functional OPA against other serotypes, including those not included in the vaccines [5,48,62]; the presence of such functional opsonic antibodies against a cross-reactive serotype may be a surrogate marker for cross-protection. Inhibition of opsonic capacity by purified capsular polysaccharides is additionally used to assess the specificity of OPA responses against cross-reactive serotypes [45,63].

ELISA and OPA antibody responses generally correlate for vaccine-specific serotypes in vaccinated individuals, but this correlation is weaker for cross-reactive serotypes not included in the vaccine. For example, one clinical trial compared the immunogenicity and safety of PCV7-CRM (containing serotype 19F, which can be cross-reactive to 19A) and PCV13-CRM (which contains serotypes 19A and 19F) in infants and toddlers in the United States [64]. There was a poor correlation between serotype 19A OPA and immunoglobulin G (IgG) responses after PCV7-CRM vaccination [64], whereas OPA immune responses after PCV13-CRM vaccination correlated with IgG responses for both serotypes 19A and 19F [64]. Similarly, another study, which was conducted in Korean children, revealed that vaccination with PCV7-CRM induced antibodies against serotype 19A that were detectable by ELISA, but limited OPA titers were observed [56]. PCV7-CRM and PCV10-mixed induced comparable levels of IgG responses against cross-reactive serotype 19A with low sero-response rates; however, OPA responses after the infant series and after the toddler booster were higher for PCV10-mixed than PCV7-CRM, raising the possibility that it might provide some clinical protection for serotype 19A [65,66,67].

As demonstrated above, there are many challenges in assessing cross-reactivity and cross-protection. In addition, studies using molecular subtyping techniques have demonstrated that *S. pneumoniae* can evolve through microevolution during an acute infection [68]. Cross-reactive antibodies may bind to pneumococcal capsular polysaccharides that are structurally related but with insufficient strength of the antigen–antibody interaction (avidity) to provide functional activity.

Therefore, an evidence-based understanding of cross-reactivity and cross-protection is valuable in the design and development of new vaccines and in understanding the potential effectiveness against serotype-specific disease. Despite challenges, cross-opsonization stands as the most reliable surrogate of cross-protection [48]. It can also provide insights for evaluating the overall effects and impacts of a vaccine, thereby informing the development of additional serotyping and surveillance activities. However, as the real-world experience with serogroup 6 and serogroup 19 cross-reactive responses has taught vaccine developers, scientists, and policymakers, clinical efficacy and effectiveness data remain the gold standard for assessing how cross-reactivity translates into cross-protection against disease and carriage.

## 5. From Past to Present: The Role of Cross-Reactivity and Cross-Protection in Pneumococcal Vaccine Design

Over the years, several pneumococcal vaccines have been developed to protect against pneumococcal disease in children and adults (Table 1) [69]. In 1977, a pneumococcal vaccine containing 14 capsular polysaccharides (PPSV14), including serotypes 1, 2, 3, 4, 6A, 7F, 8, 9N, 12F, 14, 18C, 19F, 23F, and 25F, was licensed for use in the United States, with the intent that the serotypes included in the vaccine would be sufficiently cross-protective against other serotypes within their serogroups [1,23,70]. However, certain serotypes in PPSV14 (e.g., serotypes 9N and 19F) that were anticipated to provide cross-protection against serotypes in the same serogroup failed to do so [1,23,70]. Consequently, in 1983, PPSV23 was developed, in which vaccine serotype composition was expanded to include serotypes 1, 2, 3, 4, 5, 6B, 7F, 8, 9N, 9V, 10A, 11A, 12F, 14, 15B, 17F, 18C, 19A, 19F, 20, 22F, 23F, and 33F based on serotype distribution patterns and evidence of cross-reactivity within additional serogroups [70,71]. Selection of the specific serotypes to be included in PPSV23 was primarily based on data from invasive isolates in North America and Europe. Moreover, serotype 6A was replaced by serotype 6B based on its enhanced stability in vaccine formulations [70]. In 2016, an observational study demonstrated that serotype 6B in PPSV23 induced cross-reactive antibodies to serotypes 6A, 6C, and 6D. Substantial variation in the levels of cross-reactivity were observed in both young adults (25–51 years of age) compared with older adults (≥65 years of age), whereby cross-reactive immune responses induced by 6B were lower for all serotypes in older adults [72].

As polysaccharide vaccines are poorly immunogenic in infants and toddlers, due to underdeveloped T-cell-independent immune mechanisms in this age group [7], PCVs were developed in which polysaccharides are conjugated to protein carriers; conjugation transforms the T-cell-independent polysaccharide vaccines to T-cell-dependent antigenic vaccines that are highly immunogenic in infants and young children < 2 years of age [8]. PCV7-CRM was first licensed in the United States in 2000 [1], targeting the seven serotypes most commonly responsible for pediatric invasive disease in that country, and was highly effective in reducing the incidence of IPD among vaccinated children while providing strong indirect protection to unvaccinated children and adults.

Over the past 20 years, immunogenicity and efficacy trials, as well as data on real-world effectiveness studies including direct and indirect protection, have enhanced our understanding of how cross-reactivity translates into cross-protection against disease and colonization. Comparisons between study results with different PCVs also elucidated which vaccine characteristics may contribute to both. The most detailed data regarding cross-protection are available for serotypes within two serogroups: serogroup 6, with serotypes 6A, 6B, 6C, and 6D, and serogroup 19 with serotypes 19A and 19F. Reviewing available immunogenicity, efficacy, and impact data for these two serogroups revealed several factors that contribute to cross-protection. Vaccine characteristics, such as similarities between the structure of the vaccine serotype and the cross-protective serotype, vaccine composition, and carrier protein choice are among those factors highlighted in the literature [5,46,47,84]. It has also become evident that the extent of cross-protection may differ for invasive disease compared with non-invasive disease, such as AOM and nasopharyngeal carriage, and that the dosing schedule may affect observed cross-protection [65]. This research has built on our understanding of how cross-reactivity, as measured by IgG versus OPA, may relate to cross-protection.

## 6. Lessons Learned from PCV Clinical and Real-World Evidence

Key studies highlighting examples of cross-reactivity and/or cross-protection associated with PCVs are highlighted in Table 2.

### 6.1. PCV7

Serotypes 6A and 19A were not included in PCV7-CRM, as it was hoped that the immunological similarities with vaccine serotypes 6B and 19F, respectively, could offer sufficient cross-protection to affect disease incidence [65]. Within serogroup 6, the polysaccharide structures of 6A and 6C differ only by one molecule, in which galactose is replaced by a glucose residue; serotypes 6B and 6D differ from serotypes 6A and 6C, respectively, due to a variance in linkage between rhamnose and ribitol (Figure 1) [44]. Within serogroup 19, serotypes 19A and 19F differ by the linkage to the α-L-rhamnose residue: α(1→2) for serotype 19F and α(1→3) for serotype 19A (Figure 1) [91].

Surveillance data and clinical efficacy studies have demonstrated cross-protection from the serotype 6B conjugate in PCV7-CRM against 6A IPD and AOM, along with substantial indirect protection [56,65,90,92,93,94]. One study in Belgium saw a significant decrease in 6A IPD, post-PCV7 introduction, among children < 5 years of age (incidence rate ratio (IRR): 0.31, 95% confidence interval (CI) 0.09–0.94) (Table 2) [90]. Similarly, non-significant reductions in 6A-related IPD were observed in children < 2 years of age, several years after full PCV7 implementation in the Netherlands, France, and Germany [95,96,97]. The efficacy of PCV7-CRM against serotype 6A AOM, while notable, seemed to be lower than that observed against serotype 6B (57% vs. 84%) [46], suggesting that the addition of serotype 6A into the vaccine formulation could provide additional protection against 6A AOM. Of note, with regard to nasopharyngeal colonization, Dagan et al. showed that three primary doses of PCV7-CRM (at 2, 4, and 6 months of age) substantially decreased both nasopharyngeal colonization of serotypes 6B and 6A as measured in the second year of life, while two primary doses alone (at 4 and 6 months of age) decreased colonization with vaccine serotype 6B but not cross-reactive serotype 6A [65].

When serotypes 6C and 6D were discovered in 2007 and 2010, respectively [44,98], cross-protection from serotype 6B was not anticipated for serotype 6C, based on structural dissimilarities [5,44,45,99]. In addition, the discovery of serotype 6C was important in understanding serotype replacement following the introduction of PCV7, as the apparent increase in serotype 6A incidence was subsequently determined to be attributable to the closely related serotype 6C. In a study conducted in Korean children, PCV7-CRM elicited a cross-reactive immune response against serotypes 6A, 6C, and 6D following a booster dose (opsonic activities were 100%, 78%, and 89%, respectively); however, the sample size was small [100]. By contrast, PCV7-CRM had little-to-no impact on serotype 6C disease [5,45,101,102,103].

The specific conjugate chemistry used in a particular vaccine may contribute to cross-reactivity and cross-protection. This is evident in the different outcomes reported for serotype 6A cross-protection elicited from two pneumococcal heptavalent conjugate vaccines conjugated to CRM_197_ (PCV7-CRM) or to meningococcal outer membrane protein complex (PCV7-PncOMPC) [46,47,101]. In cases of AOM, vaccine efficacy for PCV7-CRM and PCV7-PncOMPC against non-vaccine serotype 6A was 57% and −17%, respectively [46,47]. A randomized controlled trial evaluating the effectiveness of the vaccine candidate 11Pn-PD, in which serotype 6B was linked to protein D of non-typeable *Haemophilus influenzae*, indicated potential cross-protection of serotype 6B against 6A AOM [104].

In addition, despite the multiple but modest indications of vaccine efficacy and effectiveness of PCV7-CRM (containing serotype 19F) against serotype 19A IPD and AOM in clinical and post-licensure studies, the incidence of serotype 19A disease increased, subsequent to the introduction of PCV7-CRM into immunization programs. One explanation for this could be that, because serotype 19F in PCV7-CRM does not decrease 19A nasopharyngeal carriage, any direct cross-protection it might provide against 19A IPD and AOM is negated by substantial increases in 19A carriage seen in individuals vaccinated with PCV7-CRM [65].

Differences in reactive and cross-opsonic profiles against serotype 19A in infants were observed between PCV7, using the outer membrane protein of *Neisseria meningitidis* as a carrier protein, and two different formulations of an experimental five-valent PCV (PCV5 containing polysaccharides conjugated to CRM_197_ and PCV5 containing oligosaccharides to CRM_197_). All three vaccines contained serotype 19F and no 19A. In this case, the PCV7 formulation generated cross-opsonic antibodies against serotype 19A, whereas the two PCV5 formulations did not. Of note, both PCV5 formulations induced IgG antibodies against 19A as measured by ELISA, whereas PCV7 did not; the correlation between OPA and IgG antibodies for non-vaccine serotype 19A was weak as compared with vaccine-type 19F [84].

### 6.2. PCV10

PCV10-mixed was first licensed in Europe in 2009 [16] to provide protection against additional disease-causing serotypes. Evidence from population-based studies suggested that PCV10-mixed, with its inclusion of serotype 19F, provided some cross-protection against serotype 19A, but the evidence varied [105]. Two studies conducted in Brazil, using either a case–control or indirect cohort design, demonstrated substantial effectiveness against serotype 19A IPD (82% and 71%, respectively) [106,107]. Similarly, case–control studies conducted in Quebec and Finland also reported effectiveness against serotype 19A IPD (Table 2) [89,108]. Yet, in another surveillance study in the Netherlands, the effectiveness of PCV10-mixed against serotype 19A IPD failed to achieve statistical significance, with an estimate of 28% (95% CI: −179 to 81) [109].

When assessing vaccine efficacy across non-vaccine serotypes associated with IPD, a qualitative (and quantitative) difference in the cross-reactive immune responses against serotype 19A was observed between PCV7-CRM and PCV10-mixed, despite both vaccines incorporating the 19F antigen [33]. One potential explanation for this difference concerns the chemical processes used to link polysaccharides to proteins; PCV10-mixed utilizes cyanylation-mediated conjugation for binding 19F to a diphtheria toxoid carrier protein, aiming to maintain the integrity of the 19F epitope, whereas PCV7-CRM utilizes reductive amination to attach 19F to a CRM_197_ carrier, which might alter 19F epitopes by disrupting a saccharide ring [33]. Another example of differences in cross-protection based on disease outcomes can be found in the case of PCV10-mixed. While randomized controlled studies and surveillance have shown the limited ability of PCV10-mixed to prevent serotype 19A carriage, they have also shown noticeable protection against 19A-related pneumococcal disease across various regions, including areas with high serotype 19A prevalence, such as Canada (Quebec). However, heterogeneity in vaccine schedules across regions may affect observed trends [110]. As noted above, vaccine effectiveness provided by PCV10-mixed against non-vaccine serotype 19A IPD ranged from 62% to 82%, which is similar to the level of direct protection offered by PCV13-CRM against serotype 19A IPD (86%) [106,108,111,112]. However, in 2016, an increase in nasopharyngeal carriage of serotype 19A was observed after the national infant PCV program in Belgium switched from PCV13-CRM to PCV10-mixed, indicating an insufficient impact on 19A carriage through cross-reactive antibodies [113,114]. This increase was associated with an emergence of IPD attributed to serotype 19A thought to be due to distinct strains that are easily spread. As demonstrated, serotype 19F in PCV10-mixed provides moderate protection against 19A-related disease once colonized; however, it does not decrease 19A nasopharyngeal carriage. Thus, during periods of high serotype 19A infection rates, such as those observed in Belgium, PCV10-mixed seems insufficient to provide any cross-protection against serotype 19A IPD [113,114].

Of note, an increase in serotype 6C was also seen, which may be due to the absence of serotype 6A in PCV10-mixed, as compared with PCV13-CRM [113,114]. Based on these findings, PCV13-CRM was reinstated in Belgium in 2019. Similar observations were made in New Zealand after a switch from PCV13-CRM to PCV10-mixed in 2017 [115].

### 6.3. PCV13

As both PCV7-CRM and PCV10-mixed showed minimal effects on serotype 19A carriage [116], and based on the additional protection that may be offered by including serotype 6A as previously suggested by Yu et al. [84], PCV13-CRM was introduced in 2010, potentially expanding serotype coverage to include serotypes 3, 6A, and 19A [10,11]. PCV13-CRM showed strong evidence of cross-protection to serotype 6C, due to structural similarities between serotypes 6A and 6C [5,87,88,117,118,119,120,121]. The prevalence of disease due to serotype 6C decreased in the vaccinated pediatric population to a similar degree as the prevalence of disease due to serotype 6A following the introduction of PCV13 into immunization programs. However, serotype 6C remains more prevalent in adult populations, suggesting a differential impact of cross-reactive immune responses on direct versus indirect protection [27,122]. A recent systematic review also supported the impact of PCV13 on cross-protection against serotype 6C. Assessment of observational studies and randomized controlled trials revealed vaccine effectiveness against serotype 6C IPD, in the range of 70–85%, in children who had received ≥1 dose of PCV13. In addition, the prevalence of serotype 6C IPD and nasopharyngeal carriage decreased post-PCV13 introduction in most studies in children (n = 5/6) and in half of studies in adults (n = 5/11). Compared with PCV10, PCV13 vaccination protected against serotype 6C IPD and nasopharyngeal carriage in children. However, the evidence for indirect protection in adults was not consistent across studies, as serotype 6C carriage prevalence and disease incidence increased in some regions and decreased in others post-PCV13 introduction. This heterogeneity may be due to different increasing 6C serotype trends or a lack of significant cross-protection provided by 6A in PCV13, to prevent 6C carriage. Owing to the inclusion of observational studies, these results may have been subject to bias. To mitigate the impact of potential biases, the systematic review reported adjusted vaccine effectiveness and incidence rate ratios, where available [123].

### 6.4. PCV15 and PCV20

In 2021, PCV15-CRM and PCV20-CRM were also approved and recommended [12,13]. PCV15 contains serotypes 6A and 6B and elicited cross-opsonic antibodies against serotype 6C in OPA inhibition studies (Table 2) [63]; however, there are no clinical efficacy data yet available to confirm cross-protection, owing to the more recent development of the vaccine. Serotypes 6A and 6B are also included in PCV20, with a similar intent of exerting cross-protection against 6C. An exploratory analysis investigating PCV20 in infants demonstrated cross-reactive IgG and cross-functional OPA responses to serotypes 6C and 15C (Table 2) [86]. In addition, both vaccines also include serotypes 19A and 19F. Monitoring of disease epidemiology following PCV20 introduction is required to assess whether demonstrated cross-reactivity translates to cross-protection for these serotypes.

### 6.5. PCV21

In 2024, PCV21-CRM was approved in the United States for the immunization of adults ≥ 18 years of age [14]. PCV21 is population-specific in that it predominantly contains serotypes associated with the majority of IPD in adults living in countries with established pediatric vaccination programs. Before the coronavirus disease 2019 (COVID-19) pandemic, data from the United States in 2019 highlighted that serotypes included in PCV21 cause around 85% of IPD in adults ≥ 65 years of age. Of these serotypes, 30% were not included in any previously licensed vaccine (15A, 16F, 23A, 23B, 24F, 31, 35B, and deOAc15B) [83].

Early clinical studies have demonstrated post-vaccination cross-reactive immune responses to serotypes 6C and 15B from serotypes 6A and 15C included in PCV21, respectively (Table 2). A recent phase III study made similar observations, suggesting that PCV21 has the potential for cross-protection against serotype 15B. However, cross-reactive immune responses to serotype 6C provided by 6A did not meet prespecified criteria (Table 2) [85]. However, historic data suggest that, based on inclusion in other PCVs, serotype 6A can provide cross-protection against 6C disease in a real-world setting, although this is yet to be demonstrated with PCV21 vaccination [122]. Additional clinical trials in individuals who have previously received a pneumococcal vaccine, are at risk, or who are immunocompromised have been conducted, although evidence of cross-protection in these patient populations is yet to be established [85,124,125,126,127].

### 6.6. Investigational Vaccines

Other higher valent PCVs, as well as serotype-independent vaccines in early clinical development, including pneumococcal protein-based vaccines, may provide broad coverage through cross-protection, as demonstrated in preclinical and early clinical studies [7,128].

## 7. What Is Known about Cross-Reactivity and Cross-Protection from Pneumococcal Vaccines against Other Serotypes?

### 7.1. Serotypes 9A/9N/9L/9V

Serogroup 9 comprises two pairs of highly related serotypes, 9A/9V and 9L/9N. The death in 1939 of Prince Valdemar of Denmark from pneumococcal pneumonia led to the later identification of a new serotype in serogroup 9, as he was unresponsive to the available antisera 9L and 9N treatments; this serotype was posthumously named 9V in his honor [129]. Serotype 9N, which was included in PPSV14, did not provide cross-protection against serotype 9V [70]; therefore, serotype 9V was added to PPSV23 to provide additional protection against serogroup 9 pneumococcal infections [70].

As serotype 9V is more common in infants than in adults, only serotype 9V was subsequently included in PCVs primarily developed for pediatric populations [70]. There is no conclusive evidence of cross-protection from serotype 9V (included in current PCVs) to serotype 9N. A 21-valent PCV, PCV21-CRM (which includes eight unique serotypes: 15A, 15C, 16F, 23A, 23B, 24F, 31, and 35B), is targeted to adults and includes serotype 9N due to the increased prevalence of this serotype in adults compared with infants [83].

### 7.2. Serotypes 15A/15B/15C/15D/15F

Pneumococcal serogroup 15 has five members, namely serotypes 15A, 15B, 15C, 15D, and 15F. Following PCV13-CRM introduction, serotypes 15A, 15B, and 15C have become among the most prevalent serotypes associated with IPD and AOM [27,130]. Recently, a putative new serotype designated as 15D was discovered by Pimenta et al. and was described as highly related to serotypes 15A and 15F but serologically distinct based on its unique reactivity with serogroup 15 serotyping factors [131]. The structures of these four serotypes are shown in Figure 1.

The capsular polysaccharides of serotypes 15A, 15B, and 15C are closely related, and 15B is the O-acetylated version of 15C [132]. Whole-genome sequencing can now differentiate between serotypes 15B and 15C, allowing for more precise information to be captured in surveillance systems used for serotype tracking [62], but is not yet used across all surveillance systems. Both serotypes 15B and 15C are known to interconvert at high rates and are commonly reported as a pair (15B/C) in surveillance systems [133,134].

Low cross-reactivity against serotype 15C was reported for serotype 15B (included in PPSV23) based on a small number of vaccinated individuals (n = 7) [135]. However, a more extensive analysis of sera from immunized individuals (n = 28) revealed that antibodies induced by PPSV23 opsonized vaccine serotype 15B only marginally better than cross-reactive serotype 15C (1.6-fold). As serotype 15C exhibits minimal expression of the O-acetyl group, these data suggest that a PCV containing serotype 15B polysaccharide may generate antibodies that target not only the O-acetyl group but also exhibit reactivity towards the core structure of the capsular polysaccharide [134]. As noted above, a molecular modeling simulation study predicted that PCV20-CRM could provide cross-reactive antibody responses from serotype 15B to 15C. Although findings showed that the titers elicited against 15C were lower than 15B, no activity was observed against serotype 15A [62]. Similarly, in preclinical evaluations in animal models, PCV21-CRM, containing a de-O-acetylated 15B antigen that is structurally the same as the serotype 15C antigen, induced cross-functional antibodies to serotype 15B [136]. A phase III study observed cross-reactive antibody responses to 15B, provided by 15C included in PCV21 [85]. Additional longitudinal real-world evidence is required to further assess any cross-protective activity for serotypes in serogroup 15.

### 7.3. Serotypes 20A/20B

All pneumococcal isolates from serogroup 20 were initially typed as serotype 20. However, it was later discovered that serotype 20 exhibited serologic variability, resulting in the identification of serotypes 20A and 20B [137]. The serotype 20A polysaccharide is composed of the previously described serotype 20 hexasaccharide repeat unit, whereas the 20B polysaccharide is composed of a novel heptasaccharide repeat unit containing an extra branched α-glucose residue (Figure 1) [138]. Genetic analysis of the subtypes revealed that serotype 20A may have arisen from a serotype 20B progenitor, following loss-of-function mutation to a glycosyltransferase gene [138].

Epidemiologic studies have found that 20A is rare but 20B is commonly found [139]. PPSV23 contains serotype 20A, which would likely provide cross-reactivity and protection against serotype 20B. Future conjugate vaccines would ideally include serotype 20B instead of 20A [139,140]. Thus, future clinical data for these investigational vaccines should help determine potential cross-protection from serotype 20B to 20A.

### 7.4. Serotypes 23A/23B/23F

Serogroup 23 includes three serotypes: 23F (which has been included in licensed pneumococcal vaccines), 23A, and 23B (Figure 1). Ravenscroft et al. recently determined the structure of serotype 23A and showed it has a disaccharide backbone (β-rhamnose-β-glucose) with a di-substituted β-galactose linked to β-rhamnose as a side chain. Serotype 23B has the same trisaccharide backbone (β-galactose-β-rhamnose-β-glucose) as 23F but lacks the immunodominant α-rhamnose side chain (Figure 1). These structures can explain previously observed slight typing cross-reactions of serotype 23F with 23A, and no cross-reactions with serotype 23B [141].

Serotypes 23A and 23B are emerging serotypes associated with a substantial burden of pneumococcal disease in both children and adults [130,142]. The considerable increase in these serotypes is noteworthy. In the pre-vaccine era, serotypes 23A and 23B were rarely identified; only after the widespread introduction of PCVs have these serotypes emerged and become prominent in circulation. This underscores the dynamic nature of serotype epidemiology in response to vaccine (and antibiotic) selective pressure and emphasizes the crucial need to proactively evaluate vaccine candidates for cross-opsonic activity, even against currently less prevalent types. There is limited cross-reactivity from serotype 23F to 23A and no cross-reactivity with 23B. Both serotypes 23A and 23B are included in PCV21, based on epidemiology data on the most prevalent serotypes from regions with establish pediatric vaccination programs [136,143].

### 7.5. Emerging Serotypes

As PCVs continue to advance, it is possible that previously unknown cross-reactive serotypes might emerge and become clinically relevant. A recent study found that, despite the structural and antigenic similarities with serotype 33F included in PPSV23, PCV15, and PCV20, cross-protection was not observed from serotype 33F to 33E [32]. Consequently, it is crucial to evaluate vaccines to determine their capability to target and opsonize additional serotypes.

## 8. Expert Analysis and Conclusions

The effectiveness of pneumococcal vaccines to prevent both pneumococcal carriage and invasive disease, particularly in children and in high-risk adults, has been well documented [69]. However, vaccine use has also caused changes in the distribution of pneumococcal serotypes in *S. pneumoniae* through serotype replacement or capsular switching [68]. This has resulted in an increased necessity for serotyping of *S. pneumoniae* to allow monitoring and identification of the specific serotypes present in a population.

As discussed in this review, some vaccine antigens can induce immune responses that are cross-reactive, a subset of which are also cross-protective. Multiple post-marketing surveillance studies conducted following PCV introduction have helped us to confirm and extend observations seen in laboratory and controlled studies regarding cross-protection with specific types. For example, serotype 6A disease was efficiently prevented by two licensed conjugate formulations containing only 6B (PCV7-CRM and PCV10-mixed) although not another, unlicensed 6B-containing vaccine candidate (PCV7-OMP), calling into question the necessity of including serotype 6A in new, higher-valent formulations that build on the licensed vaccines to prevent 6A disease [90,92,93,94,105]. Nonetheless, the available evidence suggests that the inclusion of a serotype 6A conjugate in PCV13-CRM has led to extensive cross-protection against serotype 6C, at least in vaccinated individuals [5,45].

By contrast, the extensive cross-reactivity against serotype 19A observed with two very different 19F conjugates (in PCV7-CRM and PCV10-mixed) and cross-protection demonstrated in controlled clinical studies, even in some observational studies, did not translate into decreased serotype 19A disease incidence that was maintained over time following the introduction of either vaccine into infant immunization programs [65,89,105,106,107,108,109]. These observations and analyses of other serotypes suggest that, when selecting serotypes within epidemiologically important serogroups (such as 9, 15, 20, 23, and 33) to be directly targeted by a new vaccine, developers of PCVs need to consider laboratory assessments of cross-reactivity, especially functional assays. As this review has discussed, cross-reactive functional and cross-opsonic antibodies may be better correlated with cross-protection than total cross-reactive IgG.

In addition, those that recommend and procure vaccines will need to evaluate the same information when estimating coverage of the most prevalent disease-causing serotypes in their region. They will also need to consider that cross-protection provided by the same cross-reactive vaccine serotype can vary greatly between different PCV formulations. Cross-reactivity cannot be extrapolated across vaccines and needs to be assessed for each PCV. Lastly, it is crucial to empirically demonstrate cross-protection rather than inferring effectiveness against cross-reactive serotypes solely based on vaccine serotype composition or numbers of serotypes included in a formulation. Thus, only clinical studies that measure both disease and nasopharyngeal carriage endpoints can validate the true cross-protection afforded by a given formulation.

Marketing approval criteria for adult pneumococcal vaccines include assessing functional antibody levels measured by OPA against vaccine serotypes, which serve as a surrogate marker for efficacy against vaccine serotypes [144]. OPA should be routinely included as a secondary descriptive endpoint in pediatric trials as well, as it could help in assessing the extent of cross-protection expected in children. Furthermore, the increasing utilization of real-world evidence is poised to improve our understanding of how cross-reactivity translates to cross-protection and subsequent vaccine impact, with important implications for vaccine developers, regulatory agencies, and immunization committees.

In summary, despite the widespread use of pneumococcal vaccines and the resulting decrease in disease burden, serotype epidemiology remains dynamic, and pneumococcal disease burden persists. As the number of serotypes targeted by new PCVs increases, policymakers may favor the formulations that contain the greatest number of serotypes. However, given the differences in vaccine formulation providing cross-protection against epidemiologically important serotypes, this will be an insufficient metric. It seems inevitable that there will continue to be surprises regarding the relative importance of cross-protection among antigenically related pneumococcal serotypes.

## Figures and Tables

**Table 1:** Overview of currently available pneumococcal vaccines.

Vaccine Name	Marketed Name	Approval Year	Age Indication	Serotypes Included in Vaccine	Adjuvant	Carrier Protein	References
Pneumococcal conjugate vaccines (PCVs)							
PCV7	Prevnar^®^ Prevenar^®^	2000	≥2 months	4, 6B, 9V, 14, 18C, 19F, and 23F	Aluminum phosphate	CRM_197_	[73,74]
PCV10	Synflorix™	2009	≥6 weeks–5 years	1, 4, 5, 6B, 7F, 9V, 14, 18C, 19F, and 23F	Aluminum phosphate	Protein D, except for 18 C (tetanus toxoid) and 19F (diphtheria toxoid)	[16,75,76]
PCV10	Pneumosil^®^	2019	≥6 weeks–2 years	1, 5, 6A, 6B, 7F, 9V, 14, 19A, 19F, and 23F	Aluminum phosphate	CRM_197_	[15,17]
PCV13	Prevnar 13^©^ Prevenar 13^®^	2010	≥6 weeks	1, 3, 4, 5, 6A, 6B, 7F, 9V, 14, 19A, 19F, 18C, and 23F	Aluminum phosphate	CRM_197_	[73,77]
PCV15 (V114)	Vaxneuvance™	2021 2022	≥18 years ≥6 weeks	1, 3, 4, 5, 6A, 6B, 7F, 9V, 14, 18C, 19A, 19F, 22F, 23F, and 33F	Aluminum phosphate	CRM_197_	[78,79,80]
PCV20	Apexxnar^®^ Prevnar 20^©^ Prevenar 20^®^	2021 2023	≥18 years ≥6 weeks	1, 3, 4, 5, 6A, 6B, 7F, 8, 9V, 10A, 11A, 12F, 14, 15B, 18C, 19A, 19F, 22F, 23F, and 33F	Aluminum phosphate	CRM_197_	[81,82]
PCV21 (V116)	Capvaxive™	2024	≥18 years	3, 6A, 7F, 8, 9N, 10A, 11A, 12F, 15A, 15B, 15C, 16F, 17F, 19A, 20A, 22F, 23A, 23B, 24F, 31, 33F, and 35B	N/A	CRM_197_	[14,83]
Pneumococcal polysaccharide vaccine (PPSV)							
PPSV23	Pneumovax 23^®^	1983	≥50 years ≥2 years with increased risk of PD	1, 2, 3, 4, 5, 6B, 7F, 8, 9N, 9V, 10A, 11A, 12F, 14, 15B, 17F, 18C, 19F, 19A, 20, 22F, 23F, and 33F	N/A	N/A	[71]

Note that this is not an exhaustive list of licensed PCVs and does not include local manufacturers. CRM, cross-reacting material; PCV, pneumococcal conjugate vaccine; PCV7, 7-valent PCV; PCV10, 10-valent PCV; PCV13, 13-valent PCV; PCV20, 20-valent PCV; PD, pneumococcal disease; PPSV, pneumococcal polysaccharide vaccine; PPSV23, 23-valent pneumococcal polysaccharide vaccine; V114, 15-valent PCV.

**Table 2:** Clinical and real-world evidence for cross-reactivity of PCV-induced antibody responses.

Study	PCV	Study Design	Region	Serotype(s) of Interest *	Evidence	Limitations Reported
Cross-reactivity						
Platt H et al., 2024 [85]	PCV21	Phase III (n = 2663 adults)	Multi- national (11 countries)	6C, 15B	• ST 15C elicited cross-reactivity to ST 15B (≥four-fold rise in OPA titers in 64.7% of participants from Day 1–Day 30) • cross-reactive immune responses against ST 6C provided by ST 6A did not meet acceptable predefined criteria	• Low proportion of older adults (>75 years of age) • Did not include previously vaccinated individuals • Short study duration did not allow for assessment of NPC
Tamimi N et al., 2023 [86]	PCV20	RCT sub-analysis (n = 397 infants)	Multi- national	6C, 15C	• ST 6A elicits cross-reactive IgG and OPA responses to ST 6C, similar to those of PCV13 • ST 15B elicits cross-reactive IgG and OPA responses to ST 15C	• Small sample size • Exploratory assessment meaning analyses were descriptive and not powered
Shi Y et al., 2023 [63]	PCV15	Post-hoc analysis (n = 150 infants, n = 250 adults)	–	6C	• ST 6A induced cross-reactive antibodies to ST 6C in adult and pediatric populations comparable to those induced by PCV13	• Small sample size • Pre-absorption tests only performed in adult cohort
Cooper D, et al. 2011 [5]	PCV13	RCT sub-analysis (n = 52 infants)	Germany	6C, 7A	• ST 6A provides cross-reactive OPA responses to 6C (96% positive OPA titer; Pearson correlation of *r* = 0.78) • ST 7F provides cross-reactive OPA responses to 7A (Pearson correlation of *r* = 0.61)	• Small sample size
Lee H et al., 2009 [56]	PCV7	Observational (n = 31 infants)	Republic of Korea	6A, 19A	• ST 6B provided good levels of cross-reactivity to ST 6A (opsonic index = 81%) • ST 19F provided low levels of cross-reactivity to ST 19A (opsonic index = 19%)	• Potential inaccuracy with use of ELISA assay to measure response
Cross-protection						
Andrews N et al., 2019 [87]	PCV13	Indirect cohort (n = 3421 children)	England	6C	• ST 6A provided high levels of cross-protection against ST 6C (adjusted VE of 70.0% with ≥1 dose)	• Interpretation of VE subject to serotype trends • Long-term protection not assessed and could differ due to waning of immune response
Ahmed S et al., 2020 [88]	PCV13	Surveillance (n = 7640 adults > 19 years of age)	United States	6C	• Levels of ST 6C IPD decreased among healthy adults • Levels of ST 6C IPD remained the same among adults with comorbidities	• Reporting bias from two different surveillance systems
Rinta-Kokko H et al., 2020 [89]	PCV10	Full and indirect cohort and case control study (n = 150 children 6–102 months of age)	Finland	19A	• ST 19A provides some level of cross-protection against ST 19A (VE of 50.1% from 2010–2018)	• Subject to collection of information bias
Hanquet G et al., 2011 [90]	PCV7	Surveillance (n = 1144 children < 5 years of age)	Belgium	6A	• Significant decreases in 6A IPD were identified post-PCV7 introduction (IRR: 0.31, 95% CI 0.09–0.94)	• Reporting bias from two different surveillance systems

* Serotypes of interest include serotypes not included in the vaccine for which cross-reactivity/cross-protection was observed. Note that this table is not an exhaustive list of studies demonstrating cross-reactivity and/or cross-protection observed with all PCVs. CI, confidence interval; IgG, immunoglobulin G; IPD, invasive pneumococcal disease; IRR, incidence rate ratio; NPC, nasopharyngeal carriage; OPA, opsonophagocytic activity; PCV, pneumococcal conjugate vaccine; PCV7, 7-valent PCV; PCV10, 10-valent PCV; PCV13, 13-valent PCV; PCV15, 15-valent PCV; PCV20, 20-valent PCV; PCV21, 21-valent PCV; RCT, randomized controlled trial; ST, serotype; VE, vaccine effectiveness.
