# Correction: Feemster et al. Implications of Cross-Reactivity and Cross-Protection for Pneumococcal Vaccine Development. *Vaccines* 2024, *12*, 974

**DOI:** 10.3390/vaccines13080831

**Published:** 2025-08-04

**Authors:** Kristen Feemster, William P. Hausdorff, Natalie Banniettis, Heather Platt, Priscilla Velentgas, Alejandra Esteves-Jaramillo, Robert L. Burton, Moon H. Nahm, Ulrike K. Buchwald

**Affiliations:** 1 Merck & Co., Inc., Rahway, NJ 07065, USA; natalie.banniettis@merck.com (N.B.); heather.platt@merck.com (H.P.); priscilla.velentgas@merck.com (P.V.); alex.esteves@merck.com (A.E.-J.); ulrike.buchwald@merck.com (U.K.B.); 2 Center for Vaccine Innovation and Access, PATH, 455 Massachusetts Ave NW, Washington, DC 20001, USA; whausdorff@path.org; 3 Faculty of Medicine, Université Libre de Bruxelles, 1050 Brussels, Belgium; 4 SunFire Biotechnologies LLC, Birmingham, AL 35203, USA; rob@sunfirebio.com; 5 Heersink School of Medicine, University of Alabama at Birmingham, Birmingham, AL 35233, USA; mnahm@uabmc.edu

The authors would like to make the following corrections to this published paper [1].

Due to typographic errors, there were some mistakes in the original publication, cited above. Specifically, they are as follows: 

In Table 1, the “Approval Year” for “PVC10” (Synflorix™) should be changed from 2011 to 2009.

In Table 2, the “Region” for “PCV7” in the study by Lee H. et al., 2009 should be changed from “Belgium” to “Republic of Korea”; the year of the study by Andrews N., et al., cited as reference 87, should be changed from 2018 to 2019; the “Study Design” from Rinta-Kokko H. et al., 2020, cited as reference 89, should be changed from “Case-control study” to “Full and indirect cohort and case control study”.

In the fourth paragraph of the sub-Section 6.1 “PCV7”, the “−10%” in the sentence “In cases of AOM, vaccine efficacy for PCV7-CRM and PCV7-PncOMPC against non-vaccine serotype 6A was 57% and −10%, respectively [46,47]” should be changed to −17%.

In the first paragraph of sub-Section 6.5 “PCV21”, the last reference cited should be changed from reference 124 to reference 83, which is more suitable. 

In the second paragraph of sub-Section 7.1 “Serotypes 9A/9N/9L/9V”, the last reference cited should be changed from reference 122 to reference 83, which is more suitable.

In the second paragraph of sub-Section 7.4 “Serotypes 23A/23B/23F”, “23F” in the last sentence “Both serotypes 23A and 23F are included in PCV21, based on epidemiology data on the most prevalent serotypes from regions with establish pediatric vaccination programs [124,137]” should be changed to “23B”.

With these corrections, the order of references has been adjusted accordingly. The authors state that the scientific conclusions are unaffected. This correction was approved by the Academic Editor. The original publication has also been updated.

## References

[1] Feemster K., Hausdorff W.P., Banniettis N., Platt H., Velentgas P., Esteves-Jaramillo A., Burton R.L., Nahm M.H., Buchwald U.K. (2024). Implications of Cross-Reactivity and Cross-Protection for Pneumococcal Vaccine Development. Vaccines.

